# I want to break free: Expression of matrix metalloproteinases is necessary for cell hatching in *Chlamydomonas reinhardtii*

**DOI:** 10.1093/plcell/koae263

**Published:** 2024-09-25

**Authors:** Mariana Schuster

**Affiliations:** Assistant features editor, The Plant Cell, American Society of Plant Biologists; Leibnitz Institute of Plant Biochemistry, Receptor Biochemistry Group, Halle (Saale) 06120, Germany

*Chlamydomonas reinhardtii* (Chlamydomonas) is a unicellular green alga found in temperate soil habitats (Sasso et al. 2018). This alga displays both plant and ancestral eukaryotic features and is a powerful model organism due to its simple life cycle, convenient isolation of mutants, and expanding set of tools and methods for molecular genetics research. When resources are abundant, Chlamydomonas reproduces asexually through mitosis. As with many green algae, Chlamydomonas undergoes multiple fission during mitosis. The daughter cells are trapped within the parental cell wall until they develop new cilia and are released (hatched) when the parental cell wall breaks down (Salomé and Merchant 2019).

The cell wall in land plants and algae is part of an extracellular matrix (ECM) made up of numerous proteins and cell wall constituents. In contrast to land plants, the Chlamydomonas cell wall lacks cellulose and is composed of arabinose, mannose, galactose, and glycoproteins. It was previously described that, during hatching, the cell wall of the parental cell of Chlamydomonas is degraded by so-called vegetative and gamete lytic enzymes, such as matrix metalloproteinases (MMPs) and subtilisin-like serine proteases (Kubo et al. 2009; Zou and Bozhkov 2021), but the regulation of this process remains largely unknown. In this issue, **Minjae Kim and colleagues (Kim et al. 2024)** describe a player in Chlamydomonas that regulates cell wall degradation following mitosis. This is a kinase in the dual-specificity tyrosine phosphorylation-regulated kinase (DYRK) family, conserved across all eukaryotes. Since this kinase is found only in plant lineage, it is called plant DYRK (DYRKP1). The authors show that Chlamydomonas DYRKP1 activates the expression of MMPs, enzymes responsible for the breakdown of ECM components, thereby facilitating cell wall remodeling (Fig.) The study highlights the involvement of DYRKP1 in postmitotic cell wall dynamics, potentially providing insight into broader mechanisms of cell division and structural reorganization in plant cells.

**Figure.**
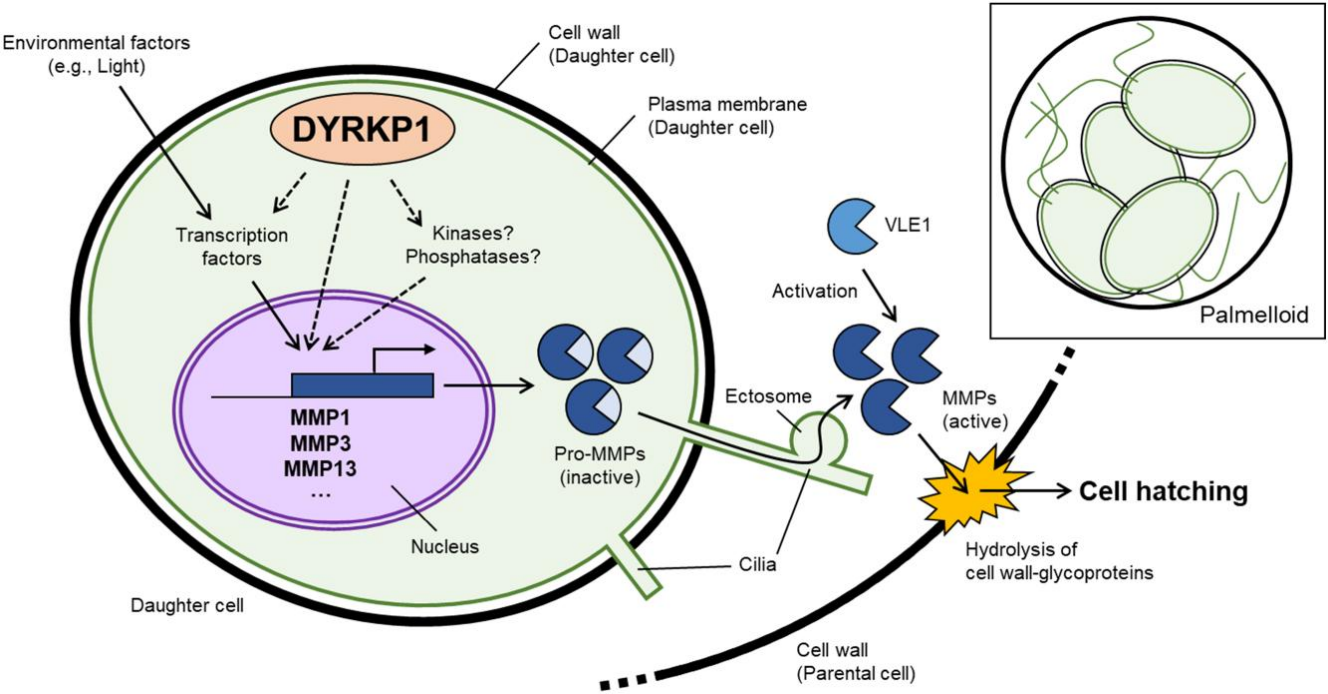
DYRKP1-induced expression of matrix MMP is required for cell hatching. DYRKP1 induces the expression of genes encoding for the matrix MMP1, MMP3, and MMP13, which are produced in an inactive form. These pro-enzymes are transported out of the cell through cilia and then activated by other proteases like vegetative lytic enzyme (VLE1). Once activated, the MMPs degrade the cell wall, allowing new cells to be released (cell hatching) and continue breaking down the remaining parts of the parental cell wall. Adapted from Kim et al. 2024, Figure 7.

The authors observed that the *dyrkp1-1* mutant, generated in a previous study, was generally larger than the parental strain (Schulz-Raffelt et al. 2016). Closer observation revealed that this was due to the formation of so-called palmelloids, large structures that encase multiple cells inside a cell wall. Moreover, in this mutant, palmelloid formation was due to a slower hatching process from the parental cell after mitosis compared with the control. Microscopic observations along with quantification of the extracellular protein in the culture medium hinted toward delayed degradation of the parental cell wall in *dyrkp1-1* compared with the parental strain. To test this observation, Kim and colleagues generated *dyrkp1* mutants in cell wall–defective Chlamydomonas strains. These mutants did not present palmelloids, supporting the hypothesis that DYRKP1 is involved in cell wall degradation. Comparative proteomics analysis between *dyrkp1-1* and the corresponding cell-walled parental strain showed that *dyrkp1-1* secretes a lower amount of cell wall proteins compared with the control.

Transcriptomic analysis corroborated that many ECM proteases were downregulated in the *dyrkp1-1* mutant. Validation of these results via RT-qPCR narrowed down the list of differentially expressed genes to *MMP1*, *MMP3*, and *MMP13*. Minjae Kim and colleagues investigated knockout mutants of MMP1, MMP3, and MMP13 and reported that these also share a palmelloid phenotype with *dyrkp1-1*. With this work, the authors have identified DYRKP1 as a regulator of cell cycle progression, which presents intriguing new opportunities for investigating the regulation of cellular processes in plants.

## Data Availability

The data underlying this article are available in the highlighted article.

## Dive Curated Terms

The following phenotypic, genotypic, and functional terms are of significance to the work described in this paper:

MMP Gramene: AT1G70170
MMP Araport: AT1G70170
